# Convection Enhanced Delivery for Diffuse Intrinsic Pontine Glioma: Review of a Single Institution Experience

**DOI:** 10.3390/pharmaceutics12070660

**Published:** 2020-07-14

**Authors:** Umberto Tosi, Mark Souweidane

**Affiliations:** 1 Department of Neurological Surgery, Weill Cornell Medicine, New York, NY 10065, USA; umt9001@nyp.org; 2 Department of Neurological Surgery, Memorial Sloan Kettering Cancer Center, New York, NY 10065, USA

**Keywords:** convection enhanced delivery (CED), diffuse intrinsic pontine glioma (DIPG), diffuse midline glioma, clinical translation

## Abstract

Diffuse intrinsic pontine gliomas (DIPGs) are a pontine subtype of diffuse midline gliomas (DMGs), primary central nervous system (CNS) tumors of childhood that carry a terrible prognosis. Because of the highly infiltrative growth pattern and the anatomical position, cytoreductive surgery is not an option. An initial response to radiation therapy is invariably followed by recurrence; mortality occurs approximately 11 months after diagnosis. The development of novel therapeutics with great preclinical promise has been hindered by the tightly regulated blood–brain barrier (BBB), which segregates the tumor comportment from the systemic circulation. One possible solution to this obstacle is the use of convection enhanced delivery (CED), a local delivery strategy that bypasses the BBB by direct infusion into the tumor through a small caliber cannula. We have recently shown CED to be safe in children with DIPG (NCT01502917). In this review, we discuss our experience with CED, its advantages, and technical advancements that are occurring in the field. We also highlight hurdles that will likely need to be overcome in demonstrating clinical benefit with this therapeutic strategy.

## 1. Diffuse Midline Gliomas: The Landscape

Pediatric diffuse midline gliomas (DMGs) are tumors of childhood with universally poor prognoses; despite accounting for only 10–20% of all pediatric central nervous system (CNS) malignancies, they are the most prevalent cause of death due to brain cancer in children [1,2]. Diffuse intrinsic pontine gliomas (DIPGs) are a particularly aggressive, inoperable subtype, characterized by an infiltrative process in the brainstem. Histologically, DIPGs are astrocytomas. They peak at 6–9 years of age, more rarely occurring in adolescents and adults. Median survival (MS) following initial radiation therapy (XRT) is 11 months; overall survival (OS) at 1 year is 30%, 10% at 2 years, with only occasional reports of survival 5 years following diagnosis [3,4]. Such a poor prognosis has not changed for decades. Usually, patients present with a triad of worsening pontine symptoms (cranial nerve deficits, cerebellar symptoms, and long tract signs) and a characteristic magnetic-resonance image (MRI), leading to diagnosis [5]. XRT (typically, 54 Gy over 6 weeks) is, currently, the only therapy to improve progression-free survival (PFS), albeit for a limited time [1,6]. DIPGs can spread along fiber tracts, with the thalamus and the cerebellum as the most common sites [7,8,9]. Spread along these tracts is believed to be carried out by cells that diverged early during tumor development and subsequently abandoned their niche, thus being strikingly different from the cells that constitute the bulk of the tumor, potentially predisposing it to drug resistance and disease recurrence [10].

The challenges to the successful treatment of DIPG are numerous. Firstly, cytoreductive surgery, an important positive predictor in most primary CNS neoplasms, is not possible, owing to highly infiltrative growth patterns and the anatomical position within the pontine segment of the brain stem. Historically, biopsies were thought to be of little benefit and have high morbidity [11]. This resulted in low tissue availability, which hindered the creation of easily-reproducible molecular and genetic profiles of DIPG, along with the establishment of solid preclinical models. In recent years, however, the use of minimally-morbid stereotactic biopsies has allowed for the establishment of such models, allowing for research to gain further traction [2,11,12,13]. A recent meta-analysis found a diagnostic success of 96.1%; an overall morbidity of 6.7%; a permanent morbidity of 0.6%, and mortality of 0.6%, supporting the use of such a procedure for diagnostic and molecular-characterization purposes [12].

Conventional and targeted therapeutic agents (Table 1) have had little to no success to date [4,14,15]. Temozolomide (TMZ), given both concurrently and after XRT, failed at achieving any survival benefit and resulted in significant toxicities [14,15]. Coupling TMZ with other therapies, such as cis-retinoic acid [16] or thalidomide [17], was similarly unsuccessful. Such a fate was shared by other traditional chemotherapeutic regimens; not only did therapies fail at prolonging survival, but they often resulted in the development of significant hematologic side effects [18,19,20,21,22]. Thus far, only one study with methotrexate, bis(2-chloroethyl)-1-nitrosourea (BCNU), cisplatin, and tamoxifen given before RDT, which was then used when disease progression occurred, has shown promise, increasing the time to XRT [23]. Dosage varied across cycles, starting at 12 mg/m^2^, 40 mg/m^2^, 40 mg/m^2^, and 20 mg/kg/d for each drug, respectively. The authors found that median survival was longer than their historical control (17 months vs. 9 months; *p* = 0.022); there was no difference, however, when the time from RDT was considered, meaning that the benefit observed was due to the chemotherapy-mediated delay in disease progression. The authors, however, also note the common development of grade III and IV complications and an overall longer length of hospital stay for patients on chemotherapy. The development of significant toxicities excluded this regimen from current clinical practice.

Targeted therapies in the treatment of DIPG have, thus far, met a similarly unsuccessful fate riddled with poor responses and significant toxicities. Tamoxifen (estrogen-receptor modifier) [31,32], bevacizumab (vascular endothelial growth factor-VEGF-inhibitor) [29], nimotuzumab (monoclonal antibody inhibitor of epidermal growth factor receptor-EGFR) [24], gefitinib (small molecule EGFR inhibitor) [30], erlotinib (EGFR inhibitor) [27], and dasatinib (BCR-Abl kinase and platelet-derived growth factor receptor A (PDGFRA) inhibitor) [25], all failed despite significant preclinical promise. Timid pre-clinical promise now comes from immunoreactive agents such as monoclonal antibodies and chimeric T cells (CAR-T cells). These agents are currently being investigated and hold particular potential because of the high tumor selectivity (leaving most non-tumor tissue unaffected) and the ability to target non-dividing cells that often escape traditional chemotherapy regimens that target highly replicating cells [33]. Most of these agents, however, are still in early phase clinical trials and their efficacy remains to be further elucidated [34,35,36,37]. High levels of tumor heterogeneity could further compound this problem, with non-reactive cells being unaffected by therapy. CAR-T cells that, for instance, target disialoganglioside GD2, a highly and almost uniformly expressed protein by H3.3K27M-mutant gliomas, have shown promise in preclinical experiments following systemic administration. However, cells with low GD2 expression escape CAR-T cells therapy. Furthermore, treatment-associated inflammation can result in hydrocephalus. Albeit a rare occurrence, such an event has been lethal in pre-clinical models [38]. The poor understanding of DIPG’s cell populations—due, in part, to scarce tumor sampling—might undermine further clinical efforts.

Given the paucity of tissue, and the overall low disease incidence, the rise of collaborative efforts aimed at changing disease prognosis has been one of the few positive notes of recent decades. Starting in the 2000s, not-for-profit foundations have been sponsoring DIPG research, resulting in the foundation of the DIPG Collaborative and its affiliated DIPG Registry—a comprehensive database of clinical, radiological, pathologic, and molecular data [39,40]. These efforts were sponsored not only by governmental funding agencies, but also—and to a great extent—by private donors. Thanks to these efforts, DIPG research and funding continue to grow with the development of new promising preclinical models, resulting in the discovery of promising novel pathways and of new therapeutics to target them [3,34,35,36,41].

These concerted efforts have allowed for the discovery of important novel DIPG features that, hopefully, will yield accessible therapeutic targets. For instance, it was determined that DIPG has an erratic epigenetic profile, with a majority of samples harboring a H3K27M mutation which, by altering histone proteins, impairs polycomb repressive complex 2 (PRC2) methyltransferase, leading to a global hypomethylation of H3K27 [3]. Such an unstable epigenetic profile makes DIPG cells particularly susceptible to histone deacetylase complex inhibitors (HDACi), compounds which have shown preclinical promise and are now being investigated for further clinical translation. Similarly, another large study determined how patients harboring the H3K27M mutation have an overall worse prognosis and shorter survival than their wildtype counterparts [41]. Albeit mutation-specific therapies are only in developmental stages, such prognostic information is of essential value and could explain, at least in part, the variable survival observed in patients. Overall, these significant findings which shed light on DIPG’s prognosis and possible therapy would not have been possible without concerted efforts by numerous specialists and funding from both private and governmental agencies.

## 2. The Development of Convection-Enhanced Delivery for Diffuse Intrinsic Pontine Glioma

One of the main issues with systemic chemotherapeutics is the inaccessibility of the tumor compartment via traditional, systemic routes because of the tightly regulated blood–brain barrier (BBB) and the blood-tumor barrier (BTB). The BBB impedes drug CNS penetration via endothelial tight junctions [42]. Traditionally, to overcome this issue, drug dosage has been increased to potentiate greater amounts of tumor penetration. This, however, often achieved only sub-therapeutic levels because of the development of dose-limiting toxicities [42]. The BBB is disrupted in the context of advanced brain tumors [43], but not in DIPG, where most of the tumor is insulated by an intact BBB, ensuring drug inaccessibility via systemic routes [6,42]. In silico methods are used to identify drugs that will theoretically cross the BBB; however, the algorithms used have thus far failed in systemic trials, with hundreds of clinical trials in DIPG not meeting endpoints despite preclinical promise, where systemic toxicity commonly impedes dose escalation to therapeutic levels [44]. Other methods to bypass the BBB include increasing drug dosage, BBB transient disruption via chemical (mannitol [45]) or mechanical (ultrasound [46]) means, intra-arterial delivery, the use of carriers, and local delivery. In BBB disruption techniques, lack of spatial sensitivity, lack of tumor specificity, or significant toxicity are problematic, and thus far efforts in DIPG have been limited due to the development of CNS toxicity [47]. Intra-arterial therapy relies on agent extraction from the circulation by the tumor on first pass; it does not circumvent BBB impermeability directly. DIPG’s pontine location and lack of a direct and single arterial feeder (as it is the case for choroid plexus tumors) weaken the case for intra-arterial therapy, thus explaining the lack of literature in children with DIPG [48]. Carriers can also be designed to cross the BBB; thus far, however, these agents lack tumor-targeting properties that would localize their distribution in DIPG, causing the development of significant CNS toxicities [49]. Local delivery strategies thus hold the most promise.

**Figure 1:**
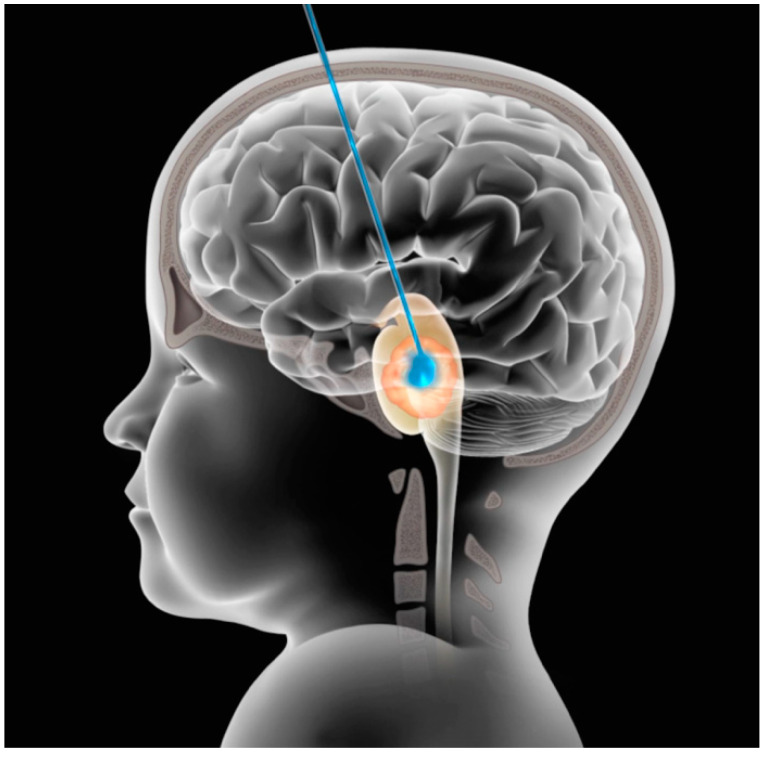
Schematics of CED. Scheme showing the supratentorial (transfrontal) approach for catheter insertion in DIPG in the pons. Adapted with permission from [39].

Convection-enhanced delivery (CED) is a technique that relies on direct cannula implantation into the brain or tumor for delivery of an infusate through a pressure gradient and could overcome these issues by locally delivering high drug concentration while minimizing systemic exposure and hence toxicity (Figure 1) [49]. The technique has been developed for at least the last two decades, both in preclinical animal models [50,51] and, more recently, in early phase clinical studies in the treatment of primary or recurrent high grade gliomas [44,51,52,53].

CED has numerous other advantages when compared to systemic delivery of a drug. For instance, CED relies on a pressure gradient to deliver its infusate, whereby so long as the pressure of delivery (P_D_) is superior to the pressure of tissue (P_T_), delivery will continue at high concentrations. On the other hand, when P_D_ < P_T_, a steep drop in concentration will occur. The almost-constant concentration where P_D_ > P_T_ allows for the even permeation of homogeneous tissue and guarantees a more favorable spatial profile, i.e., allows for coverage of a greater volume at high concentrations than simple diffusion, which decays exponentially.

Recent developments in catheter design allow for the creation of different infusions profile, as each catheter tries to address a specific problem associated with the use of CED in vivo. For instance, a stepped profile catheter, with a reduced gauge at the end, tries to minimize backflow in the needle tract (a low-pressure sink), known to be directly proportional to catheter gauge. Multiport cannulas reduce the turbulent flow observed at the end of end-port cannulas, where high velocities can reduce overall volume of distribution, with flow being turbulent rather than linear. Porous tipped catheter (similar to multiport catheters, but with more numerous and smaller (≤0.45 µm diameter) holes) increase the distribution of infusate into the surrounding gel substrate and murine brain tissue. Balloon tipped catheters, albeit rarely used for CED, have been employed in the treatment of post-resection cavities for maximal permeation of the tumor penumbra [54]. Numerous questions about the efficacy of CED, however, remain.

Catheter backflow is another significant hindrance to CED [49]. The needle tract represents a low pressure sink with low hydraulic resistance when compared to normal brain that favors infusate flow, resulting in lower permeation of target tissue [55]. Different solutions to this problem exist; as mentioned, a stepped catheter can reduce backflow by diminishing the caliber of the tract. Allowing for sealing time (i.e., for the brain to adhere to the catheter) is another solution, along with the use of a lower infusion rate: a smaller pressure differential will result, diminishing the relevance of a pressure sink [55]. Furthermore, a rapid needle insertion speed reduces backflow, as tissue damage is limited (favoring brain sealing on the needle) [50]. Despite the numerous studies that have looked at backflow and the qualitative description of some of the properties that seem to favor or reduce it, a careful mathematical model that predicts it is still lacking.

DIPG is a logical tumor prototype for using a CED therapeutic backbone for several reasons. Relative to other infiltrative gliomas, DIPG is more constrained within a limited anatomical compartment, meaning that representative drug distribution should be more easily achieved. The avoidance of cytoreductive surgical tumor removal leaves the tumor milieu without gross inhomogeneities, a desirable feature for CED. Metastatic tumor dissemination is known to occur in DIPG but typically not early in the disease continuum. Lastly, the urgent need for innovative therapeutic strategies for a universally fatal disease provides a lesser threshold for regulatory approval for unconventional strategies like CED. Balancing these appealing features early on were the predictable intolerance of the brain stem to stressors, the use of an otherwise unnecessary surgical procedure, and an uncertain clinical risk profile. To date, a sample of clinical studies have demonstrated the safety and feasibility of this approach via injection of a variety of agents (e.g., IL13-Pseudomonas toxin or the oncolytic virus delta-24-RGD); however, all these studies had great variability in technique, infusate, and hardware used, making it difficult to draw unifying conclusions about their efficacy and on the role of each independent variable assessed [56,57,58,59,60,61].

**Figure 2:**
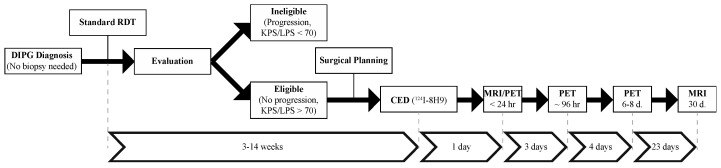
Workflow of NCT01502917. CED was performed before recurrence following radiation. No biopsy was necessary for enrollment. Monitoring was carried out with both magnetic-resonance image (MRI) and positron emission tomography (PET).

Given the variability in technique and hence the lack of existing data to define safe or meaningful parameters related to CED in the brain stem, we designed a Phase 1 clinical trial (NCT01502917) that would for the first time use an iterative dose, volume, and rate escalation design. Children with a clinical and radiographic diagnosis of nonprogressive DIPG who underwent standard radiation therapy were eligible for enrollment (Figure 2). CED of the radiolabeled monoclonal antibody Omburtamab (y-mAbs Therapeutics; New York, NY, USA) was then performed between 4–14 weeks following XRT. Post-operative serial monitoring was performed with MRI (post-treatment days 1 and 30) and positron emission tomography (PET) (post-treatment days 1, 4, 7). The primary objective of this trial was to assess the safety of CED in children with DIPG and to define safe infusion parameters (radionuclide dose, infusion rate, and infusion volume). Unlike preceding the DIPG experience, the intervention was carried out before disease progression and did not require a confirmatory biopsy for enrollment.

^124^I-Omburtamab is a monoclonal antibody that targets the membrane-bound protein CD276 (B7-H3), an immune modulator part of the B7 superfamily overexpressed in DIPG and other pediatric CNS cancers [62,63,64]. Its role in disease development and progression is still under investigation [62,65]; however, the overexpression in tumor tissue, especially when compared to normal brain parenchyma, made it a very appealing therapeutic target. This feature is found in DIPG as well as other pediatric malignancies [64]. In addition to the therapeutic potential, the radionuclide affords quantitative PET imaging, making it a true theranostic molecule with both therapeutic and imaging potential.

NCT01502917 heavily relied on treatment planning and on the use of novel technologies developed or re-purposed for CED. Once in the operating room, a semi-flexible catheter (BrainLab, Munich, Germany) (used under an investigational exemption) was then inserted under MR guidance using a fully MR-compatible interface (Clear Point Neuro, Irvine, CA, USA). Infusion of ^124^I-8H9 (Omburtamab) was carried out at the bedside, with the patient in an intensive care unit environment. Post-operative and post-infusion imaging were carried out as schematized in Figure 2 [37]. Infusion volumes were ranged from 240 μL to 4540 μL, for a prescribed dose from 0.25 mCi to 4.00 mCi.

The trial is still ongoing; as of May 2020, toxicities observed include left-sided muscle weakness, ataxia, and dysarthria (all Grade III) in one patient, and generalized muscle weakness (Grade III) in another. These add to the previously reported transient Grade III hemiparesis and one Grade III skin infection [37]. No Grade IV treatment-related adverse event occurred. Based on our work thus far, the average volume of distribution to volume of infusion (Vd/Vi) ratio is 3.4 ± 1.2, which means that for our highest published single infusion volume dose level of 4000 μL, distribution volumes of approximately 12 mL are expected. Of note, the average lesion-to-whole body absorbed dose ratio is in excess of 1000, indicating that very high intralesional doses are attained with negligible systemic exposure. This is nearly an inverse to what is expected with systemic drug delivery where only a small fraction of the drug reaches the CNS parenchymal compartment [37].

Since our initial experience [37], continued work has demonstrated the safety of CED in treating children with DIPG with volumes of infusions greater than 8000 μL with Vd up to 35 mL, thus allowing for coverage of most post-radiation tumor volumes, and showed the potential of this approach that could be easily expanded to include other therapeutics that are poor BBB penetrators.

Our experience using CED in children with DIPG has allayed many of the concerns related to feasibility and safety of this therapeutic platform. The eventual objective, however, is to demonstrate disease response and clinical benefit. As of May 2020, the children treated on this clinical protocol thus far have a median overall survival rate of 17.5 months with 58.5% survival at 1 year (as previously published), which surpasses that of most cooperative group trials [66]. Furthermore, hope comes from children who have demonstrated long-term survival (three surviving more than 3 years and one now 6 years post-treatment) with seemingly radiographic local control. Making any definitive statements related to survival benefit or potential prognostic variable based on our Phase 1 experience remains difficult. The variability in patients at the time of treatment, tumor burden, prescribed dose, distribution volume, absorbed dose, and tumor coverage is notable. Tumor volume (Vtum), for instance, has high variability and could easily impact outcome. We have shown how determining tumor volume in the case of DIPG is a challenge, with high degrees of inter-observer variability [67]. This is due, at least in part, to the diffuse infiltrative nature of the tumor and lack of clear Gd-DTPA enhancement, which make defining a border difficult. In our case, Vtum ranged between 5 and 60 mL. Additionally, volumes of infusion (Vi) have now ranged from 240 to 8000 μL, with volumes of distribution up to 35 mL.

## 3. Optimizing Convection Enhanced Delivery for DIPG

In our study, Vd was estimated as a change in T2-weighted signal on the post-infusion MRI compared to the baseline pretreatment MRI. Based on our patients treated thus far we have observed a Vd/Vi ratio of approximately 3, which is consistent with other available preclinical and clinical experience [37,52,56,68,69,70]. The use of a true theranostic agent, ^124^I radionuclide, in our clinical experience has also provided an additional methodology for measuring Vd. Based on our PET-based analytics there is a positive linear relationship between Vd and Vi, but the slope of that line (Vd/Vi) is less than 3.

What also remains unknown is the longitudinal behavior of distribution. Others have shown how during and immediately after CED, T2-weighted changes can approximate actual infusate distribution, consistently with the biophysics of CED [71,72]. It remains unclear, however, if such a co-distribution remains after convection is finished [73]. In our case, PET signal could be detected in the brainstem up to a week following injection; T2 changes, on the other hand, resolved more rapidly. Nonetheless, immediately after injection, we noticed good concordance between the distribution volumes approximated with T2-weighted MRI and PET thresholds of approximately 30%. The optimal threshold for PET is now the subject of ongoing analysis.

The use of PET has evident advantages over the delivery of non-imageable agents. However, the number of therapeutics that can be directly imaged is limited. Nonetheless, recent developments in synthetic radiochemistry have expanded the library of compounds that can be modified and transformed into theranostics, maintaining the original compound’s bioactivity. For instance, we have experience in generating [^124^I]- and [^18^F]-labeled agents [74,75,76,77,78,79,80]. Of note, the isotope conjugated with the molecule will have great impact on its fate and clinical applicability. Whereas [^124^I] (half-life of 4.2 days) allows for more accurate long-term tracking of drug behavior, the rapid decay of [^18^F] (half-life of 109.7 min), which reduces ionizing radiation to a patient, and its ready availability in cancer centers that use fludeoxyglucose for tumor mapping, make [^18^F] a preferred isotope for translation, especially if a patient must receive multiple drug doses. Novel key aqueous radiochemistry allows to avoid the use of protective groups during radiosynthesis, thus making the process easier, more rapid, and non-interfering with the molecule’s original binding pocket [81,82]. This allows to achieve single-step, direct [^18^F]–[^19^F] PET isotopic exchange radiolabeling of drug molecules that bear complex functionality. Agents such as antibodies and peptides have long in vivo half-lives, making [^124^I] more suitable for their long-term imaging.

Logically, maximal tumor coverage by the administered therapeutic compound is a desired goal. Simply, there should be at least complete overlap between the Vtum and Vd. Using that criterion as a measure of successful drug administration, our estimations of drug–tumor intersect reveal large variance, ranging between 25% and 96%. Logically, smaller Vtum and greater Vi would both lead to greater Vd and hence tumor intersect. In addition, enhanced targeting to avoid longitudinal white matter tracts, pial or ependymal surfaces, and necrotic/cystic regions will undoubtedly play a role in optimizing tumor coverage. In future trials aimed at demonstrating clinical benefit, degree of tumor coverage will be monitored for any importance of outcome.

## 4. Measuring and Predicting Tumor Coverage

**Figure 3:**
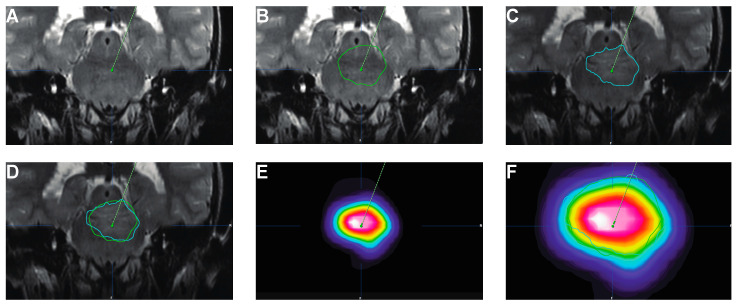
Examples of Predictive software for Vd. T2-weighted MRIs showing (**A**) actual catheter positioning prior to infusion; (**B**) predicted Vd before infusion (green outline; 9.8 mL); (**C**) actual Vd following infusion determined as ΔT2 (blue outline; 9.6 mL); (**D**) overlap between predicted (green) and actual (blue) Vd. (**E**) PET image showing actual tracer distribution following delivery. (**F**) Magnified PET image showing actual tracer distribution following delivery projected over predicted Vd (green) and actual Vd (ΔT2, blue). Images courtesy of Eva Wembacher, BrainLab (Munich, Germany).

It is essential to maximize tumor coverage with the therapeutic infusate given, both over space and time. Failure to do so could result in the development of resistance and, eventually, tumor recurrence [83,84,85]. A possible solution to optimize overlap between Vtum and Vd is the use of simulation software that predicts Vd based on tissue imaging characteristics and infusion parameters [86,87]. Numerous hurdles need to be overcome for these methods to be readily used in the clinic; however, since their adoption, we have observed significant concordance between the predicted Vd and the actual Vd determined with either PET or MRI (Figure 3). The use of theranostic agents will further allow to verify the validity of such software via direct imaging.

A second issue pertains to the behavior of infusates following CED; since convection relies on the establishment of a pressure gradient between infusion front and brain parenchyma, infusates are most likely to fall into low pressure wells, such as tumor cystic components or catheter tract (backflow). To overcome these issues, new step catheters are being developed to reduce backflow [88,89,90,91,92] or to optimize reflux to cover structures along the tract [58]. New implantable devices with single or multiple catheters are also being developed to allow for re-dosing or continuous infusions, maximizing the exposure time to the therapeutic given [56,69,70,93]. One of the main challenges of implantable systems, besides the possible safety concerns associated with foreign bodies, is the fact that, if a first infusion were to be effective, a second one, in the same target, would hardly be beneficial, as it would occur in (ideally) necrotic tissue. In our experience, patients have also been re-dosed a second or third time with ^124^I-8H9, tolerating the procedure well; however, each intervention required a new surgery [37].

These issues are compounded by the lack of accurate and non-invasive drug monitoring tools. With the exception of radiolabeled agents as ^124^I-8H9, the distributive volume and pharmacokinetics profile of most infused chemotherapeutics cannot be readily visualized—monitoring tools like CSF sampling or tissue biopsy are burdensome and carry significant morbidity. Co-infused agents, albeit effective at approximating distributive features at time 0, are not reliable longitudinally [73]. For this reason, theranostics, agents with both imaging and therapeutic properties, are being developed for CED, based either on modifications of small molecules [79,94,95] or relying on larger carriers [49,75,96,97]. Albeit promising, these agents are yet to be translated to the clinic successfully; their use, however, will guide dosing in early phase clinical trials to maximize therapeutic potential.

## 5. The Paradox of Using CED for DIPG

CED has the potential of achieving high regional drug concentrations while limiting overall body exposure to a therapeutic. It remains unclear, however, if such an approach could be curative for DIPG. In fact, various recent studies have shown how, at the time of autopsy, up to a third of patients had leptomeningeal disease spread and a fourth had disease outside the brainstem [7]. Other series found how, at the time of autopsy, more than half of the samples had leptomeningeal disease, with a third of them presenting, at the time of diagnosis, with such a finding [8]. Dissemination to other CNS sites has also been observed and associated with a worse prognosis [9]. Clonal analysis of pontine and extra-pontine tumor samples also revealed how migration out of the brainstem occurred early during disease progression, possibly prior to diagnosis and XRT [10].

It is clear, therefore, how CED could achieve regional disease control but fail at covering other distant areas. Nonetheless, it holds the potential for controlling brainstem pathology and, if coupled with other innovative approaches, such as craniospinal radiation or intrathecal delivery of chemotherapeutics, could change, at least in part, the dire prognosis [98]. The use of CED to effectively deliver chemotherapeutic agents (chemosurgery) would thus become one of the weapons in the arsenal against DIPG.

The best agent (or combination of agents) to be given via CED is still matter of debate; however, the last few years have seen an increase in CED-based clinical trials for DIPG; besides ^124^I-8H9 (NCT01502917) [37], IL13-Pseudomonas toxin (NCT00880061), MTX-110 (a water soluble version of the histone deacetylase complex inhibitor Panobinostat; NCT03566199), and DNX-2401 (an oncolytic virus; NCT03178032) are being investigated as potential agents [57,59,60,99,100]. These clinical trials, albeit few in number, represent a significant increase from previous work, where CED was not commonly utilized for drug delivery to the brain and carry the hope of changing such an unfavorable prognosis.

## 6. Conclusions

Our experience shows how CED is a safe technique in treating DIPG and, if further developed, could hopefully achieve local tumor control. However, numerous hurdles — ranging from further understanding of pharmacokinetics to optimization of therapeutic agent — remain to be overcome before such a goal could be realistically reached. Further, given DIPG’s behavior and early distant spread, CED will most likely be one tool among many in the arsenal necessary to tackle DIPG and change its otherwise abysmal prognosis. 

## Figures and Tables

**Table 1:** Summary of relevant conventional and targeted therapy-based clinical trials in the treatment of DIPG.

First Author	Year Published	Country	Therapy/Target	Median PFS (Months)		MS (Months)		OS at 1 Year (%)
Bailey [14]	2013	England	TMZ (long regimen)	5.6		9.5		35
Bradley [22]	2013	USA	Motexafin-gadolinium, XRT	7.2		11.4		53
Chassot [15]	2012	France	TMZ	7.5		11.7		50
Frappaz [23]	2008	France	Methotrexate, BCNU, cisplatin, tamoxifen.	N/R		17		N/R
Kim [17]	2009	S. Korea	TMZ, thalidomide	7.2		12.7		58.3
Korones [19]	2008	USA	Vincristine, VP-16	N/R		9		27
Massimino [20]	2008	Italy	Etoposide, cytarabine, ifosfamide, cisplatin, dactinomycin	7		12		45
Massimino [20]	2008	Italy	Cisplatin/etoposide, cyclophosphamide/ vincristine/methotrexate	10		13		70
Massimino [20]	2008	Italy	Cisplatin/etoposide, isotretinoin	5		9		29
Massimino [20]	2008	Italy	Vinorelbine	7		9		43
Sirachainan [16]	2008	Thailand	TMZ, cis retinoic acid	10.2		13.5		58
Wolff [21]	2006	USA	INF-γ, cyclophosphamide	7.2		9.6		N/R
Wolff [18]	2010	USA	Cisplatin, etoposide, vincristine, ifosfamide	4.8		13.6		N/R
Bartels [24]	2014	USA, Canada	Nimotuzumab/EGFR	N/R		3.2		N/R
Broniscer [25]	2013	USA	Dasatinib/PDGFRA, vandetanib/VEGFR	N/R		15.0		52
Broniscer [26]	2018	USA	Dasatinib/PDGFRA, crizotinib/c-Met	N/R, “short”		N/R		N/R
Geoerger [27]	2011	France	Erlotinib/EGFR	1.5	8.0	4.1	12.0	N/R
Michalski [28]	2010	UK	Tamoxifen/estrogen receptor	3.9		6.3		16.1
Narayana [29]	2010	USA	BEV/VEGF, Irinotecan/topoisomerase	2.25		6.25		20.0
Pollack [30]	2011	USA	Gefitinib/EGFR	7.4		N/R		56.4

## References

[1] Kebudi R., Cakir F.B. (2013). Management of diffuse pontine gliomas in children: Recent developments. Paediatr. Drugs.

[2] Panditharatna E., Yaeger K., Kilburn L.B., Packer R.J., Nazarian J. (2015). Clinicopathology of diffuse intrinsic pontine glioma and its redefined genomic and epigenomic landscape. Cancer Genet..

[3] Grasso C.S., Tang Y., Truffaux N., Berlow N.E., Liu L., Debily M.A., Quist M.J., Davis L.E., Huang E.C., Woo P.J. (2015). Functionally defined therapeutic targets in diffuse intrinsic pontine glioma. Nat. Med..

[4] Robison N.J., Kieran M.W. (2014). Diffuse intrinsic pontine glioma: A reassessment. J. Neuro Oncol..

[5] Schroeder K.M., Hoeman C.M., Becher O.J. (2014). Children are not just little adults: Recent advances in understanding of diffuse intrinsic pontine glioma biology. Pediatric Res..

[6] Warren K.E. (2012). Diffuse intrinsic pontine glioma: Poised for progress. Front. Oncol..

[7] Buczkowicz P., Bartels U., Bouffet E., Becher O., Hawkins C. (2014). Histopathological spectrum of paediatric diffuse intrinsic pontine glioma: Diagnostic and therapeutic implications. Acta Neuropathol..

[8] Sethi R., Allen J., Donahue B., Karajannis M., Gardner S., Wisoff J., Kunnakkat S., Mathew J., Zagzag D., Newman K. (2011). Prospective neuraxis mri surveillance reveals a high risk of leptomeningeal dissemination in diffuse intrinsic pontine glioma. J. Neuro Oncol..

[9] Wagner S., Benesch M., Berthold F., Gnekow A.K., Rutkowski S., Strater R., Warmuth-Metz M., Kortmann R.D., Pietsch T., Wolff J.E. (2006). Secondary dissemination in children with high-grade malignant gliomas and diffuse intrinsic pontine gliomas. Br. J. Cancer.

[10] Vinci M., Burford A., Molinari V., Kessler K., Popov S., Clarke M., Taylor K.R., Pemberton H.N., Lord C.J., Gutteridge A. (2018). Functional diversity and cooperativity between subclonal populations of pediatric glioblastoma and diffuse intrinsic pontine glioma cells. Nat. Med..

[11] Cage T.A., Samagh S.P., Mueller S., Nicolaides T., Haas-Kogan D., Prados M., Banerjee A., Auguste K.I., Gupta N. (2013). Feasibility, safety, and indications for surgical biopsy of intrinsic brainstem tumors in children. Childs Nerv. Syst..

[12] Hamisch C., Kickingereder P., Fischer M., Simon T., Ruge M.I. (2017). Update on the diagnostic value and safety of stereotactic biopsy for pediatric brainstem tumors: A systematic review and meta-analysis of 735 cases. J. Neurosurg. Pediatr..

[13] Kambhampati M., Perez J.P., Yadavilli S., Saratsis A.M., Hill A.D., Ho C.Y., Panditharatna E., Markel M., Packer R.J., Nazarian J. (2015). A standardized autopsy procurement allows for the comprehensive study of dipg biology. Oncotarget.

[14] Bailey S., Howman A., Wheatley K., Wherton D., Boota N., Pizer B., Fisher D., Kearns P., Picton S., Saran F. (2013). Diffuse intrinsic pontine glioma treated with prolonged temozolomide and radiotherapy--results of a united kingdom phase ii trial (cns 2007 04). Eur. J. Cancer.

[15] Chassot A., Canale S., Varlet P., Puget S., Roujeau T., Negretti L., Dhermain F., Rialland X., Raquin M.A., Grill J. (2012). Radiotherapy with concurrent and adjuvant temozolomide in children with newly diagnosed diffuse intrinsic pontine glioma. J. Neuro Oncol..

[16] Sirachainan N., Pakakasama S., Visudithbhan A., Chiamchanya S., Tuntiyatorn L., Dhanachai M., Laothamatas J., Hongeng S. (2008). Concurrent radiotherapy with temozolomide followed by adjuvant temozolomide and cis-retinoic acid in children with diffuse intrinsic pontine glioma. Neuro Oncol..

[17] Kim C.Y., Kim S.K., Phi J.H., Lee M.M., Kim I.A., Kim I.H., Wang K.C., Jung H.L., Lee M.J., Cho B.K. (2010). A prospective study of temozolomide plus thalidomide during and after radiation therapy for pediatric diffuse pontine gliomas: Preliminary results of the korean society for pediatric neuro-oncology study. J. Neuro Oncol..

[18] Wolff J.E., Driever P.H., Erdlenbruch B., Kortmann R.D., Rutkowski S., Pietsch T., Parker C., Metz M.W., Gnekow A., Kramm C.M. (2010). Intensive chemotherapy improves survival in pediatric high-grade glioma after gross total resection: Results of the hit-gbm-c protocol. Cancer.

[19] Korones D.N., Fisher P.G., Kretschmar C., Zhou T., Chen Z., Kepner J., Freeman C. (2008). Treatment of children with diffuse intrinsic brain stem glioma with radiotherapy, vincristine and oral vp-16: A children’s oncology group phase ii study. Pediatric Blood Cancer.

[20] Massimino M., Spreafico F., Biassoni V., Simonetti F., Riva D., Trecate G., Giombini S., Poggi G., Pecori E., Pignoli E. (2008). Diffuse pontine gliomas in children: Changing strategies, changing results? A mono-institutional 20-year experience. J. Neuro Oncol..

[21] Wolff J.E., Wagner S., Reinert C., Gnekow A., Kortmann R.D., Kuhl J., Van Gool S.W. (2006). Maintenance treatment with interferon-gamma and low-dose cyclophosphamide for pediatric high-grade glioma. J. Neuro Oncol..

[22] Bradley K.A., Zhou T., McNall-Knapp R.Y., Jakacki R.I., Levy A.S., Vezina G., Pollack I.F. (2013). Motexafin-gadolinium and involved field radiation therapy for intrinsic pontine glioma of childhood: A children’s oncology group phase 2 study. Int. J. Radiat. Oncol. Biol. Phys..

[23] Frappaz D., Schell M., Thiesse P., Marec-Berard P., Mottolese C., Perol D., Bergeron C., Philip T., Ricci A.C., Galand-Desme S. (2008). Preradiation chemotherapy may improve survival in pediatric diffuse intrinsic brainstem gliomas: Final results of bsg 98 prospective trial. Neuro Oncol..

[24] Bartels U., Wolff J., Gore L., Dunkel I., Gilheeney S., Allen J., Goldman S., Yalon M., Packer R.J., Korones D.N. (2014). Phase 2 study of safety and efficacy of nimotuzumab in pediatric patients with progressive diffuse intrinsic pontine glioma. Neuro Oncol..

[25] Broniscer A., Baker S.D., Wetmore C., Pai Panandiker A.S., Huang J., Davidoff A.M., Onar-Thomas A., Panetta J.C., Chin T.K., Merchant T.E. (2013). Phase i trial, pharmacokinetics, and pharmacodynamics of vandetanib and dasatinib in children with newly diagnosed diffuse intrinsic pontine glioma. Clin. Cancer Res..

[26] Broniscer A., Jia S., Mandrell B., Hamideh D., Huang J., Onar-Thomas A., Gajjar A., Raimondi S.C., Tatevossian R.G., Stewart C.F. (2018). Phase 1 trial, pharmacokinetics, and pharmacodynamics of dasatinib combined with crizotinib in children with recurrent or progressive high-grade and diffuse intrinsic pontine glioma. Pediatric Blood Cancer.

[27] Geoerger B., Hargrave D., Thomas F., Ndiaye A., Frappaz D., Andreiuolo F., Varlet P., Aerts I., Riccardi R., Jaspan T. (2011). Innovative therapies for children with cancer pediatric phase i study of erlotinib in brainstem glioma and relapsing/refractory brain tumors. Neuro Oncol..

[28] Michalski A., Bouffet E., Taylor R.E., Hargrave D., Walker D., Picton S., Robinson K., Pizer B., Bujkiewicz S. (2010). The addition of high-dose tamoxifen to standard radiotherapy does not improve the survival of patients with diffuse intrinsic pontine glioma. J. Neuro Oncol..

[29] Narayana A., Kunnakkat S., Chacko-Mathew J., Gardner S., Karajannis M., Raza S., Wisoff J., Weiner H., Harter D., Allen J. (2010). Bevacizumab in recurrent high-grade pediatric gliomas. Neuro Oncol..

[30] Pollack I.F., Stewart C.F., Kocak M., Poussaint T.Y., Broniscer A., Banerjee A., Douglas J.G., Kun L.E., Boyett J.M., Geyer J.R. (2011). A phase ii study of gefitinib and irradiation in children with newly diagnosed brainstem gliomas: A report from the pediatric brain tumor consortium. Neuro Oncol..

[31] Baltuch G.H., Couldwell W.T., Villemure J.G., Yong V.W. (1993). Protein kinase c inhibitors suppress cell growth in established and low-passage glioma cell lines. A comparison between staurosporine and tamoxifen. Neurosurgery.

[32] Couldwell W.T., Hinton D.R., Surnock A.A., DeGiorgio C.M., Weiner L.P., Apuzzo M.L., Masri L., Law R.E., Weiss M.H. (1996). Treatment of recurrent malignant gliomas with chronic oral high-dose tamoxifen. Clin. Cancer Res..

[33] Luther N., Zhou Z., Zanzonico P., Cheung N.K., Humm J., Edgar M.A., Souweidane M.M. (2014). The potential of theragnostic (1)(2)(4)i-8h9 convection-enhanced delivery in diffuse intrinsic pontine glioma. Neuro Oncol..

[34] Aziz-Bose R., Monje M. (2019). Diffuse intrinsic pontine glioma: Molecular landscape and emerging therapeutic targets. Curr. Opin. Oncol..

[35] Nagaraja S., Vitanza N.A., Woo P.J., Taylor K.R., Liu F., Zhang L., Li M., Meng W., Ponnuswami A., Sun W. (2017). Transcriptional dependencies in diffuse intrinsic pontine glioma. Cancer Cell.

[36] Vitanza N.A., Monje M. (2019). Diffuse intrinsic pontine glioma: From diagnosis to next-generation clinical trials. Curr. Treat. Options Neurol..

[37] Souweidane M.M., Kramer K., Pandit-Taskar N., Zhou Z., Haque S., Zanzonico P., Carrasquillo J.A., Lyashchenko S.K., Thakur S.B., Donzelli M. (2018). Convection-enhanced delivery for diffuse intrinsic pontine glioma: A single-centre, dose-escalation, phase 1 trial. Lancet. Oncol..

[38] Mount C.W., Majzner R.G., Sundaresh S., Arnold E.P., Kadapakkam M., Haile S., Labanieh L., Hulleman E., Woo P.J., Rietberg S.P. (2018). Potent antitumor efficacy of anti-gd2 car t cells in h3-k27m(+) diffuse midline gliomas. Nat. Med..

[39] Kuzan-Fischer C.M., Souweidane M.M. (2019). The intersect of neurosurgery with diffuse intrinsic pontine glioma. J. Neurosurg. Pediatr..

[40] Campbell C., Greenfield J.P. (2018). Precision oncogenomics in pediatrics: A personal reflection. Cold Spring Harb. Mol. Case Stud..

[41] Mackay A., Burford A., Carvalho D., Izquierdo E., Fazal-Salom J., Taylor K.R., Bjerke L., Clarke M., Vinci M., Nandhabalan M. (2017). Integrated molecular meta-analysis of 1,000 pediatric high-grade and diffuse intrinsic pontine glioma. Cancer Cell.

[42] Warren K.E. (2018). Beyond the blood:Brain barrier: The importance of central nervous system (cns) pharmacokinetics for the treatment of cns tumors, including diffuse intrinsic pontine glioma. Front. Oncol..

[43] Vengoji R., Ponnusamy M.P., Rachagani S., Mahapatra S., Batra S.K., Shonka N., Macha M.A. (2018). Novel therapies hijack the blood brain barrier to eradicate glioblastoma cancer stem cells. Carcinogenesis.

[44] Gwak H.S., Park H.J. (2017). Developing chemotherapy for diffuse pontine intrinsic gliomas (dipg). Crit. Rev. Oncol. Hematol..

[45] Foley C.P., Rubin D.G., Santillan A., Sondhi D., Dyke J.P., Crystal R.G., Gobin Y.P., Ballon D.J. (2014). Intra-arterial delivery of aav vectors to the mouse brain after mannitol mediated blood brain barrier disruption. J. Control. Release.

[46] Chu P.C., Chai W.Y., Tsai C.H., Kang S.T., Yeh C.K., Liu H.L. (2016). Focused ultrasound-induced blood-brain barrier opening: Association with mechanical index and cavitation index analyzed by dynamic contrast-enhanced magnetic-resonance imaging. Sci. Rep..

[47] Warren K., Jakacki R., Widemann B., Aikin A., Libucha M., Packer R., Vezina G., Reaman G., Shaw D., Krailo M. (2006). Phase ii trial of intravenous lobradimil and carboplatin in childhood brain tumors: A report from the children’s oncology group. Cancer Chemother. Pharm..

[48] Fortin D., McAllister L.D., Nesbit G., Doolittle N.D., Miner M., Hanson E.J., Neuwelt E.A. (1999). Unusual cervical spinal cord toxicity associated with intra-arterial carboplatin, intra-arterial or intravenous etoposide phosphate, and intravenous cyclophosphamide in conjunction with osmotic blood brain-barrier disruption in the vertebral artery. Ajnr. Am. J. Neuroradiol..

[49] Tosi U., Marnell C.S., Chang R., Cho W.C., Ting R., Maachani U.B., Souweidane M.M. (2017). Advances in molecular imaging of locally delivered targeted therapeutics for central nervous system tumors. Int. J. Mol. Sci..

[50] Casanova F., Carney P.R., Sarntinoranont M. (2012). Influence of needle insertion speed on backflow for convection-enhanced delivery. J. Biomech. Eng..

[51] Chittiboina P., Heiss J.D., Warren K.E., Lonser R.R. (2014). Magnetic resonance imaging properties of convective delivery in diffuse intrinsic pontine gliomas. J. Neurosurg. Pediatr..

[52] Hardy P.A., Keeley D., Schorn G., Forman E., Ai Y., Venugopalan R., Zhang Z., Bradley L.H. (2013). Convection enhanced delivery of different molecular weight tracers of gadolinium-tagged polylysine. J. Neurosci. Methods.

[53] Ivasyk I., Morgenstern P.F., Wembacher-Schroeder E., Souweidane M.M. (2017). Influence of an intratumoral cyst on drug distribution by convection-enhanced delivery: Case report. J. Neurosurg. Pediatr..

[54] Lewis O., Woolley M., Johnson D., Rosser A., Barua N.U., Bienemann A.S., Gill S.S., Evans S. (2016). Chronic, intermittent convection-enhanced delivery devices. J.Neurosci. Methods.

[55] Chen M.Y., Lonser R.R., Morrison P.F., Governale L.S., Oldfield E.H. (1999). Variables affecting convection-enhanced delivery to the striatum: A systematic examination of rate of infusion, cannula size, infusate concentration, and tissue-cannula sealing time. J. Neurosurg..

[56] Barua N.U., Lowis S.P., Woolley M., O’Sullivan S., Harrison R., Gill S.S. (2013). Robot-guided convection-enhanced delivery of carboplatin for advanced brainstem glioma. Acta Neurochir.

[57] Heiss J.D., Jamshidi A., Shah S., Martin S., Wolters P.L., Argersinger D.P., Warren K.E., Lonser R.R. (2018). Phase i trial of convection-enhanced delivery of il13-pseudomonas toxin in children with diffuse intrinsic pontine glioma. J. Neurosurg. Pediatr..

[58] Lewis O., Woolley M., Johnson D.E., Fletcher J., Fenech J., Pietrzyk M.W., Barua N.U., Bienemann A.S., Singleton W., Evans S.L. (2018). Maximising coverage of brain structures using controlled reflux, convection-enhanced delivery and the recessed step catheter. J. Neurosci. Methods.

[59] Tejada S., Alonso M., Patino A., Fueyo J., Gomez-Manzano C., Diez-Valle R. (2018). Phase i trial of dnx-2401 for diffuse intrinsic pontine glioma newly diagnosed in pediatric patients. Neurosurgery.

[60] Tejada S., Diez-Valle R., Dominguez P.D., Patino-Garcia A., Gonzalez-Huarriz M., Fueyo J., Gomez-Manzano C., Idoate M.A., Peterkin J., Alonso M.M. (2018). Dnx-2401, an oncolytic virus, for the treatment of newly diagnosed diffuse intrinsic pontine gliomas: A case report. Front. Oncol..

[61] Lonser R.R., Warren K.E., Butman J.A., Quezado Z., Robison R.A., Walbridge S., Schiffman R., Merrill M., Walker M.L., Park D.M. (2007). Real-time image-guided direct convective perfusion of intrinsic brainstem lesions. Technical note. J. Neurosurg..

[62] Baral A., Ye H.X., Jiang P.C., Yao Y., Mao Y. (2014). B7-h3 and b7-h1 expression in cerebral spinal fluid and tumor tissue correlates with the malignancy grade of glioma patients. Oncol. Lett..

[63] Maachani U.B., Tosi U., Pisapia D.J., Mukherjee S., Marnell C.S., Voronina J., Martinez D., Santi M., Dahmane N., Zhou Z. (2019). B7-h3 as a prognostic biomarker and therapeutic target in pediatric central nervous system tumors. Transl. Oncol..

[64] Zhou Z., Luther N., Ibrahim G.M., Hawkins C., Vibhakar R., Handler M.H., Souweidane M.M. (2013). B7-h3, a potential therapeutic target, is expressed in diffuse intrinsic pontine glioma. J. Neuro Oncol..

[65] Wang Z., Wang Z., Zhang C., Liu X., Li G., Liu S., Sun L., Liang J., Hu H., Liu Y. (2018). Genetic and clinical characterization of b7-h3 (cd276) expression and epigenetic regulation in diffuse brain glioma. Cancer Sci..

[66] Lu V.M., Alvi M.A., McDonald K.L., Daniels D.J. (2018). Impact of the h3k27m mutation on survival in pediatric high-grade glioma: A systematic review and meta-analysis. J. Neurosurg. Pediatr..

[67] Singh R., Zhou Z., Tisnado J., Haque S., Peck K.K., Young R.J., Tsiouris A.J., Thakur S.B., Souweidane M.M. (2016). A novel magnetic resonance imaging segmentation technique for determining diffuse intrinsic pontine glioma tumor volume. J. Neurosurg. Pediatr..

[68] Barua N.U., Hopkins K., Woolley M., O’Sullivan S., Harrison R., Edwards R.J., Bienemann A.S., Wyatt M.J., Arshad A., Gill S.S. (2016). A novel implantable catheter system with transcutaneous port for intermittent convection-enhanced delivery of carboplatin for recurrent glioblastoma. Drug Deliv..

[69] Barua N.U., Woolley M., Bienemann A.S., Johnson D.E., Lewis O., Wyatt M.J., Irving C., O’Sullivan S., Murray G., Fennelly C. (2013). Intermittent convection-enhanced delivery to the brain through a novel transcutaneous bone-anchored port. J. Neurosci. Methods.

[70] Fan X., Nelson B.D., Ai Y., Stiles D.K., Gash D.M., Hardy P.A., Zhang Z. (2015). Continuous intraputamenal convection-enhanced delivery in adult rhesus macaques. J. Neurosurg..

[71] Mehta A.I., Choi B.D., Ajay D., Raghavan R., Brady M., Friedman A.H., Pastan I., Bigner D.D., Sampson J.H. (2012). Convection enhanced delivery of macromolecules for brain tumors. Curr. Drug Discov. Technol..

[72] Sampson J.H., Brady M., Raghavan R., Mehta A.I., Friedman A.H., Reardon D.A., Petry N.A., Barboriak D.P., Wong T.Z., Zalutsky M.R. (2011). Colocalization of gadolinium-diethylene triamine pentaacetic acid with high-molecular-weight molecules after intracerebral convection-enhanced delivery in humans. Neurosurgery.

[73] Tosi U., Souweidane M.M. (2020). Longitudinal monitoring of gd-dtpa following convection enhanced delivery in the brainstem. World Neurosurg..

[74] An F.F., Kommidi H., Chen N., Ting R. (2017). A conjugate of pentamethine cyanine and (18)f as a positron emission tomography/near-infrared fluorescence probe for multimodality tumor imaging. Int. J. Mol. Sci..

[75] Bellat V., Ting R., Southard T.L., Vahdat L., Molina H., Fernandez J., Aras O., Stokol T., Law B. (2018). Functional peptide nanofibers with unique tumor targeting and enzyme-induced local retention properties. Adv. Funct. Mater..

[76] Jurgielewicz P., Harmsen S., Wei E., Bachmann M.H., Ting R., Aras O. (2017). New imaging probes to track cell fate: Reporter genes in stem cell research. Cell. Mol. Life Sci..

[77] Kommidi H., Guo H., Chen N., Kim D., He B., Wu A.P., Aras O., Ting R. (2017). An [(18)f]-positron-emitting, fluorescent, cerebrospinal fluid probe for imaging damage to the brain and spine. Theranostics.

[78] Kommidi H., Guo H., Nurili F., Vedvyas Y., Jin M.M., McClure T.D., Ehdaie B., Sayman H.B., Akin O., Aras O. (2018). (18)f-positron emitting/trimethine cyanine-fluorescent contrast for image-guided prostate cancer management. J. Med. Chem..

[79] Kommidi H., Tosi U., Maachani U.B., Guo H., Marnell C.S., Law B., Souweidane M.M., Ting R. (2018). (18)f-radiolabeled panobinostat allows for positron emission tomography guided delivery of a histone deacetylase inhibitor. Acs Med. Chem. Lett..

[80] Wang Y., An F.F., Chan M., Friedman B., Rodriguez E.A., Tsien R.Y., Aras O., Ting R. (2017). (18)f-positron-emitting/fluorescent labeled erythrocytes allow imaging of internal hemorrhage in a murine intracranial hemorrhage model. J. Cereb. Blood Flow Metab..

[81] Ting R., Aguilera T.A., Crisp J.L., Hall D.J., Eckelman W.C., Vera D.R., Tsien R.Y. (2010). Fast 18f labeling of a near-infrared fluorophore enables positron emission tomography and optical imaging of sentinel lymph nodes. Bioconjugate Chem..

[82] Ting R., Harwig C., auf dem Keller U., McCormick S., Austin P., Overall C.M., Adam M.J., Ruth T.J., Perrin D.M. (2008). Toward [18f]-labeled aryltrifluoroborate radiotracers: In vivo positron emission tomography imaging of stable aryltrifluoroborate clearance in mice. J. Am. Chem. Soc..

[83] Phillips W.T., Bao A., Brenner A.J., Goins B.A. (2014). Image-guided interventional therapy for cancer with radiotherapeutic nanoparticles. Adv. Drug Deliv. Rev..

[84] Phillips W.T., Goins B., Bao A., Vargas D., Guttierez J.E., Trevino A., Miller J.R., Henry J., Zuniga R., Vecil G. (2012). Rhenium-186 liposomes as convection-enhanced nanoparticle brachytherapy for treatment of glioblastoma. Neuro Oncol..

[85] Sampson J.H., Archer G., Pedain C., Wembacher-Schroder E., Westphal M., Kunwar S., Vogelbaum M.A., Coan A., Herndon J.E., Raghavan R. (2010). Poor drug distribution as a possible explanation for the results of the precise trial. J. Neurosurg..

[86] Rosenbluth K.H., Eschermann J.F., Mittermeyer G., Thomson R., Mittermeyer S., Bankiewicz K.S. (2012). Analysis of a simulation algorithm for direct brain drug delivery. Neuroimage.

[87] Rosenbluth K.H., Martin A.J., Mittermeyer S., Eschermann J., Dickinson P.J., Bankiewicz K.S. (2013). Rapid inverse planning for pressure-driven drug infusions in the brain. Plos ONE.

[88] Elenes E.Y., Rylander C.G., De Vleeschouwer S. (2017). Maximizing local access to therapeutic deliveries in glioblastoma. Part ii: Arborizing catheter for convection-enhanced delivery in tissue phantoms. Glioblastoma.

[89] Morgenstern P.F., Zhou Z., Wembacher-Schroder E., Cina V., Tsiouris A.J., Souweidane M.M. (2018). Clinical tolerance of corticospinal tracts in convection-enhanced delivery to the brainstem. J. Neurosurg..

[90] Prabhu S.S. (2018). Convection-enhanced delivery for management of malignant gliomas. Prog. Neurol. Surg..

[91] Stine C.A., Munson J.M. (2019). Convection-enhanced delivery: Connection to and impact of interstitial fluid flow. Front. Oncol..

[92] Lueshen E., Tangen K., Mehta A.I., Linninger A. (2017). Backflow-free catheters for efficient and safe convection-enhanced delivery of therapeutics. Med. Eng. Phys..

[93] D’Amico R.S., Neira J.A., Yun J., Alexiades N.G., Banu M., Englander Z.K., Kennedy B.C., Ung T.H., Rothrock R.J., Romanov A. (2019). Validation of an effective implantable pump-infusion system for chronic convection-enhanced delivery of intracerebral topotecan in a large animal model. J. Neurosurg..

[94] Tosi U., Kommidi H., Bellat V., Marnell C.S., Guo H., Adeuyan O., Schweitzer M.E., Chen N., Su T., Zhang G. (2019). Real-time, in vivo correlation of molecular structure with drug distribution in the brain striatum following convection enhanced delivery. ACS Chem. Neurosci..

[95] Wang M., Kommidi H., Tosi U., Guo H., Zhou Z., Schweitzer M.E., Wu L.Y., Singh R., Hou S., Law B. (2017). A murine model for quantitative, real-time evaluation of convection-enhanced delivery (rt-ced) using an (18)[f]-positron emitting, fluorescent derivative of dasatinib. Mol. Cancer Ther..

[96] Singh R., Bellat V., Wang M., Schweitzer M.E., Wu Y.L., Tung C.H., Souweidane M.M., Law B. (2018). Volume of distribution and clearance of peptide-based nanofiber after convection-enhanced delivery. J. Neurosurg..

[97] Bellat V., Lee H.H., Vahdat L., Law B. (2016). Smart nanotransformers with unique enzyme-inducible structural changes and drug release properties. Biomacromolecules.

[98] Fowler M.J., Cotter J.D., Knight B.E., Sevick-Muraca E.M., Sandberg D.I., Sirianni R.W. (2020). Intrathecal drug delivery in the era of nanomedicine. Adv. Drug Deliv. Rev..

[99] Lang F.F., Conrad C., Gomez-Manzano C., Yung W.K.A., Sawaya R., Weinberg J.S., Prabhu S.S., Rao G., Fuller G.N., Aldape K.D. (2018). Phase i study of dnx-2401 (delta-24-rgd) oncolytic adenovirus: Replication and immunotherapeutic effects in recurrent malignant glioma. J. Clin. Oncol..

[100] Singleton W.G.B., Bienemann A.S., Woolley M., Johnson D., Lewis O., Wyatt M.J., Damment S.J.P., Boulter L.J., Killick-Cole C.L., Asby D.J. (2018). The distribution, clearance, and brainstem toxicity of panobinostat administered by convection-enhanced delivery. J. Neurosurg. Pediatr..

